# Pressure ulcer image segmentation technique through synthetic frequencies generation and contrast variation using toroidal geometry

**DOI:** 10.1186/s12938-016-0298-3

**Published:** 2017-01-06

**Authors:** Ortiz P. David, Daniel Sierra-Sosa, Begoña García Zapirain

**Affiliations:** 1 Mathematical Modeling Research Group, School of Sciences, Universidad EAFIT, Carrera 49 NO 7 Sur-50, Medellín, Colombia; 2 DeustoTech - Fundación Deusto, Avda/Universidades 24, 48007 Bilbao, Spain; 3 Facultad Ingeniería, Universidad de Deusto, Avda/Universidades 24, 48007 Bilbao, Spain

**Keywords:** Image segmentation, Medical images, Synthetic frequencies, Toroidal geometry

## Abstract

**Background:**

Pressure ulcers have become subject of study in recent years due to the treatment high costs and decreased life quality from patients. These chronic wounds are related to the global life expectancy increment, being the geriatric and physical disable patients the principal affected by this condition. Injuries diagnosis and treatment usually takes weeks or even months by medical personel. Using non-invasive techniques, such as image processing techniques, it is possible to conduct an analysis from ulcers and aid in its diagnosis.

**Methods:**

This paper proposes a novel technique for image segmentation based on contrast changes by using synthetic frequencies obtained from the grayscale value available in each pixel of the image. These synthetic frequencies are calculated using the model of energy density over an electric field to describe a relation between a constant density and the image amplitude in a pixel. A toroidal geometry is used to decompose the image into different contrast levels by variating the synthetic frequencies. Then, the decomposed image is binarized applying Otsu’s threshold allowing for obtaining the contours that describe the contrast variations. Morphological operations are used to obtain the desired segment of the image.

**Results:**

The proposed technique is evaluated by synthesizing a Data Base with 51 images of pressure ulcers, provided by the Centre IGURCO. With the segmentation of these pressure ulcer images it is possible to aid in its diagnosis and treatment. To provide evidences of technique performance, digital image correlation was used as a measure, where the segments obtained using the methodology are compared with the real segments. The proposed technique is compared with two benchmarked algorithms. The results over the technique present an average correlation of 0.89 with a variation of ±0.1 and a computational time of 9.04 seconds.

**Conclusions:**

The methodology presents better segmentation results than the benchmarked algorithms using less computational time and without the need of an initial condition.

## Background

Pressure ulcers are skin injuries produced due to prolonged state of inactivity. They result due to the constriction from soft tissue between a bone prominence and an outer surface. The compression causes local occlusions of blood capillaries, decreasing blood flow and leading to skin and muscular ischemia, which degenerates in necrosis and cell death if the compression is not diminished [1, 2]. Also, the evolution of the injury is different in skin and muscle. Muscles are more prone to develop ischemia than skin, due to an anaerobic metabolism, thus, lesions first starts deep in the muscle before appearing in the skin [1].

In Europe about 18% of patients referred to hospitals develop pressure ulcers [2], from them 70% are elderly people. Also, it is reported that about 50% of patients develop the injuries in the first weeks [3]. Furthermore, patients with pressure ulcers have a decease probability related with this pathology of 25%, but with proper diagnosis and treatment an estimate of 95% of death cases are evitable [2].

In general, the average treatment costs of an ulcer in Europe ranges from  €1500 to  €17000 [2]. Countries as UK reports  €1.8 million of annual costs [4], in Spain the cost extents to  €461 millions per year [5] and for Netherlands, cost ranges from  €371 to  €1.695 millions [6]. Other studies report that pressure ulcers occur each year in the United States, with annual costs exceeding $1.3 billion and incidence of almost 15% in hospitalized patients aged 65 years or more, for a time equal to or greater than five days [7].

These wounds, depending on the state in which they are can take years to recover, causing severe pain and increasing the risk of secondary infections [8]. Injuries diagnosis and treatment usually takes weeks or even months, leading to an increment in health and social costs. The National Pressure Ulcer Advisory Panel (NPUAP) classifies the wounds in four stages [9]:

- *Stage I* Redness of the skin around the area of pressure. This stage has no loss of skin but the affected area differs from changes in skin thickness and temperature.
- *Stage II* Partial loss of skin thickness. The wound could have the appearance of a burn with reddish pink color. In addition, it can look like an intact or open/ruptured serum-filled blister.
- *Stage III* Full skin thickness loss. Subcutaneous tissues can be visible, but bone, tendon or muscles are not exposed. Necrotic tissue may be present but does not obscure the depth of tissue loss. May include tunneling (degradation in the depth of the wound).
- *Stage IV* Full skin thickness loss with exposed bone, tendon or muscle. Necrotic tissue and crust may be present on some parts of the wound. Often include tunneling.

On stage IV, the wound has the characteristic of being completely visible. Usually, the medical personnel responsible for conducting the wounds evaluation use visual methods to take measurements. The observation of the size and color of the ulcer is useful to parameterize the lesions. The inaccurate measures due to rustic methodologies difficult the treatment and in some cases, are ineffective. Using non-invasive techniques, such as taking pictures of the injury, it is possible to conduct an analysis from ulcers by using image processing techniques. By digitalizing the information of the image, it is possible to describe the characteristics of the wound, providing an alternative to the measures made by hand. One of the most common techniques is image segmentation, which is particularly important when performing the synthesis and analysis of medical images, since this technique allows for extracting sections that are studied in patients and facilitate diagnosis as presented in Kass et al. [10].

The correct segmentation of structures within the image provides useful information for eventual diagnosis. It is important to provide a technique that allows for properly discriminate the image sections, considering that they may be blurred, making them difficult to separate compromising important structures over it.

For image segmentation, the common used techniques are related to curve evolution methods because of their adaptive capacity and modeling of the internal structures of the images. By giving an initial seed, the level set method [11] and the active contour (frequently called Snakes) [12], use a variational problem for minimize an energy functional that generally depends of the gradient of the image [13, 14]. On [15] authors propose a functional that mixed the use of the gradient with the average energy of the image for stopping the evolution of the curve, giving an advantage over images were the gradient is not defined.

Other works as [16], use the combination of the watershed algorithm with seed region growing algorithm to determine the different image sections that share same characteristics and determine image segments. Also, in [17] authors propose a technique that uses a Sobel operator to get images borders, and from this, calculate thresholds that are compared with the entropy of the image to get the objects in the image. Some works as [18] use fuzzy logic to segment objects over the image that are soft and diffuse, others like [19] use that uses entropy as a fuzzy characteristic over the image. Some other techniques were developed taking advantage of the dynamic of the image. On [20] the spatial dependencies between the arrays of closed elements in magnetic resonance images are used to segment objects. In [21] local measures are introduced to make regularized spatial segmentation.

The present work proposes a segmentation technique that allows for identifying image structures efficiently from transformations over the image. To develop the segmentation, the extraction of synthetic frequencies is performed by using the model of the energy density over an electric field to describe a relation between a constant density and the image amplitude in grayscale values of a pixel, then, a toroidal geometry was employed to decompose the image over multiple contrast levels by variating the synthetic frequencies. Finally, Otsu’s method is applied to binarize the decomposed image in order to get contours that describe the contrast variation. This scheme enhances contrast at different levels allowing the blurred structures to be defined with more precision so the diagnosis is better.

The objective of this work is to develop a technique which allows for define the edges of objects within the image so that they can be separated for further analysis. To achieve this three approaches are presented: generate a framework of transform for contrast enhancement, relate the spatial information with the contrast of the images and extract particular structures over the image by the enhancement of the contrast.

The paper is organized as follows: in “Methods” the proposed methodology is presented. First, the extraction of the synthetic frequencies procedure is described, then, two operations derived from the toroidal geometry are proposed. Finally, Otsu’s thresholding method is applied to binarized the images. In “Results and analysis” a short description of the data base provided by the Center IGURCO is given and an analysis over the test and the comparison between algorithms is performed. Finally, “Conclusions” has the conclusions over the results.

## Methods

Given a RGB image, the three color channels are transformed into one single-valued monochromatic image [22] by following the linear combination

1 $$\begin{aligned} Y = 0.2126 \cdot R + 0.7152 \cdot G + 0.0722 \cdot B \end{aligned}$$

where R represents the red channel, G the green channel and B the blue channel. The image obtained from this combination is said to be in grayscale. The weighting values are defined by the CIE 1931 system, which relates the electromagnetic visible spectrum and the human physiological color perception [23].

This monochromatic image can be defined as the function $A: \Omega \subset \mathbb {R}^2 \rightarrow \mathbb {R}^+$ the purpose of image segmentation is to find closed subsets $\Omega _i \subset \Omega$ where the information in the function *A*, over those subsets, is correlated. These subsets satisfy

2 $$\begin{aligned} \Omega = \bigcup \limits _{i=1}^{N} \Omega _i \text {;} \quad \bigcap \limits _{i=1}^{N} \Omega _i^{(0)} = \emptyset \end{aligned}$$

where $\Omega _i^{(0)}$ is the interior of $\Omega _i$ and *N* is the number of subsets that constitute the segmentation. To get these subsets, a functional is formulated as a variational problem, were the segmentation goals are defined by describing how the subsets fit the most the correlated data in the image [11]. The proposed technique decomposes the image in different contrast levels in order to use them as input images for the functional. To achieve this, the technique is carried out in three steps. First, synthetic frequencies are calculated, considering digital images as the result of the intensity recording from light that impinges over each pixel, the local intensity calculated by using the energy density from the electric field on each light receptor, allowing for describing a relation between the contrast density and the image amplitude in each pixels. These pixels are assumed to have unit area and the synthetic frequencies are those decomposed from the electric field in all pixels from sensor.

Once the synthetic frequencies are defined, a toroidal geometry is used to decompose the image into different contrast levels by variating the synthetic frequencies. The toroidal geometry is selected due to the toroid symmetry, allowing for describe x and y axis as independent periodic functions setting the contrast levels on image from synthetic frequencies. Finally, Otsu’s optimization method is used as the functional to binarize the decomposed image repeating the process for each contrast level.

### Synthetic frequencies

To calculate the synthetic frequencies, it was considered the model of the energy density over an electric field defined as

3 $$\begin{aligned} \mu _\varepsilon = \frac{1}{2} c \varepsilon _0 E^2 \end{aligned}$$

where *E* is the magnitude of the energy field, $\varepsilon _0$ is the vacuum permittivity and *c* is the light speed. Over a fixed point, the plane wave equation is defined as

4 $$\begin{aligned} y(t) = A \sin (\omega t) \end{aligned}$$

where $\omega$ is the angular frequency and its derivative

5 $$\begin{aligned} c(t) = \frac{dy(t)}{dt} = A \omega \cos (\omega t). \end{aligned}$$

Replacing the wave derivative (5) in (3) and understanding the energy *E* as the amplitude *A* in each pixel, the energy density of the pixel is expressed as

6 $$\begin{aligned} \mu _\varepsilon = \frac{1}{2}A \omega \cos (\omega t) \varepsilon _0 A^2. \end{aligned}$$

Without loss of generality and to simplify the analysis, suppose that energy density will be 1 when ${\cos (\omega t)}$ gets the maximum value and replacing the angular frequency ${\omega = 2\pi f}$, (6) can be rewritten as

7 $$\begin{aligned} f = \frac{1}{\pi \varepsilon _0 A^3}. \end{aligned}$$

**Fig. 1:**
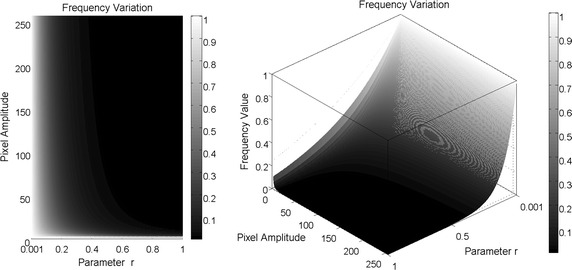
Synthetic frequencies calculated over amplitude values from 1 to 256, using variation of the parameter *r* from $10^{-3}$ to 1

As it can be seen in (7), there is an inverse relation between frequency and the amplitude of the image. As the scope of the work is to calculate synthetic frequencies using just the amplitude of the image over the pixel, (7) is simplified as

8 $$\begin{aligned} f(m,n;r) = \frac{1}{A^r(m,n)} \end{aligned}$$

where *A*(*m*, *n*) is the amplitude in gray scale of the pixel (*m*, *n*), *n* goes from 0 to $N -1$ and *m* goes from 0 to $M-1$, *N* and *M* are the number of pixels vertically and horizontally, related to the dimension of the image and ${r \in \mathbb {R}^+}$ is called the frequency parameter. The objective of variate this parameter, is to give different values to the frequency with a decreasing asymptotic behavior as it can be seen on Fig. 1.

### Image decomposition

For image decomposition, the proposed technique uses the parametric equations that define the toroidal geometry. The functions that describe the coordinate plane *xy* or the torus are redefine in order to express the torus operators of addition and product. The toroidal geometry is expressed as the parametric surface ${\vec{T}(\theta , \phi ) = (x,y,z)}$. Here, the parametric surface can be seen as the transformation ${\vec{T}: \mathbb {R}^2 \rightarrow \mathbb {R}^3}$ which means that the two input variables in the domain are transformed in a coordinate point in $\mathbb {R}^3$. The equations of the torus are

9 $$\begin{aligned} \vec{T}(\theta , \phi ) = {\left\{ \begin{array}{ll} x(\theta ,\phi ) = (R+r\cos \theta )\cos \phi &{} \\ y(\theta ,\phi ) = (R+r\cos \theta )\sin \phi &{} \quad \theta ,\phi \in [0,2\pi ]\\ z(\theta ,\phi ) = r\sin \theta \end{array}\right. } \end{aligned}$$

where *R* and *r* are the major and minor torus radius respectively. It can be seen on (9) that *x* and *y* are the mappings ${[0,2\pi ] \times [0,2\pi ] \subset \mathbb {R}^2 \rightarrow \mathbb {R}}$. As it was mentioned before, these variables are modified in order to express the torus operations. Rewriting $\theta$ and $\phi$ in (9), the new maps are defined as ${x: \mathbf {u} \rightarrow \mathbb {R}}$ and ${y: \mathbf {v} \rightarrow \mathbb {R}}$

10 $$\begin{aligned} \begin{array}{ll} x(\mathbf {u}) & = THD(\mathbf {u};f) = [R+\cos (2\pi f\mathbf {u})]\cos (2\pi \arctan (f))\\ y(\mathbf {v}) & = TVD(\mathbf {v};f) = [R+\cos (2\pi f\mathbf {v})]\sin (2\pi \arctan (f)) \end{array} \end{aligned}$$

where *TVD* means toroidal vertical decomposition, *THD* means toroidal horizontal decomposition and *f* is the synthetic frequency defined in (8). These new maps decompose the image vertically and horizontally depending of the domain definition. In this case, the domain for *x* is defined as ${\mathbf {u}=\{ \mathbf {u}_j\}^T_{j= 0 \dots N-1}}$, where ${\mathbf {u}_j= [\frac{-2m}{M} \dots \frac{2m}{M}]^T}$, ${m=0 \dots \frac{(M-1)}{2}}$ and for *y* the domain will be ${\mathbf {v} = \{\mathbf {v}_k\}_{k=0 \dots M-1}}$, where ${\mathbf {v}_k = [\frac{-2n}{N} \dots \frac{2n}{N}]^T}$, ${n=0 \dots \frac{(N - 1)}{2}}$ . The objective of this decomposition is to modify the periodicity, in the *x* or *y* axis (depending on the map), of the contrast of the image by variating the parameter *r* in (8). From (10) two operations are defined as

11 $$\begin{aligned} \begin{array}{ll} T_a(\mathbf {u},\mathbf {v};f) &{}= A(m,n) \circ [TVD(\mathbf {v};f) + THD(\mathbf {u};f)] \\ T_p(\mathbf {u},\mathbf {v};f) &{}= A(m,n)^2 \circ [TVD(\mathbf {v};f) \circ THD(\mathbf {u};f)] \end{array} \end{aligned}$$

where the product $\circ$ between matrices is the Hadamard product defined as ${(B \circ C)_{i,j} = (B)_{i,j} \cdot (C)_{i,j}}$. Finally, absolute value is obtained from each element of the operations in (11) in order to avoid negative values and the result is divided by the maximum so the decomposition is normalized

12 $$\begin{aligned} \bar{T}_a = \frac{1}{\max (|T_a|)}|T_a|; \qquad \bar{T}_p = \frac{1}{\max (|T_p|)}|T_p|. \end{aligned}$$

### Threshold

The last step in the proposed technique is the image thresholding. This step is based on Otsu optimization method for thresholding images in order to reduce the gray scale values to a binary values [24]. To obtain the binary image, the method uses the probability distribution over the gray scale values of the image to divide then in two classes, $C_0$ and $C_1$, that encloses the gray levels in the range 0 to $t-1$ and *t* to *L*, being *L* the maximum contrast level (gray levels goes from 0 to 255). To obtain the threshold *t*, the mean levels $\mu _0$ and $\mu _1$ for $C_0$ and $C_1$ can be expressed as

13 $$\begin{aligned} \mu _0 = \sum _{i=0}^{t-1} \frac{ip_i}{\omega _0(t)} \qquad \mu _1 = \sum _{i=t}^{L-1} \frac{ip_i}{\omega _1(t)} \end{aligned}$$

where $\omega _0(t)=\sum _{i=0}^{t-1} p_i$, $\omega _1(t)=\sum _{i=t}^{L-1} p_i$ and $p_i$ is the probability for the gray scale level *i*. The mean intensity $\mu _T$ is represented as

14 $$\begin{aligned} \mu _T = \omega _0 \mu _0 + \omega _1 \mu _1, \qquad \omega _0 + \omega _1 = 1. \end{aligned}$$

The objective is to found *t* that maximizes

15 $$\begin{aligned} J(t) = \sigma _0 + \sigma _1 \end{aligned}$$

where $\sigma _0 = \omega _0(\mu _0 - \mu _T)^2$ and $\sigma _1 = \omega _1(\mu _1 - \mu _T)^2$. By finding the threshold *t*, each pixel is separated in two classes depending of the amplitude in gray scale values. In this case, Otsu’s method is applied to the product matrix $\bar{T}_{pj}(m,n)$ defined in (12), where the sub-index *j* means the contrast level by variating the frequency parameter. The functional is expressed as

16 $$\begin{aligned} \max _{t_j} J(t_j; \bar{T}_{pj}). \end{aligned}$$

Finally, setting $N = 2$ for the number of subsets on (2), segmentation for each contrast level is defined as

17 $$\begin{aligned} \begin{array}{ll} \Omega _1^j &{}= \{ (m,n) | \bar{T}_a(m,n) \le t_j \}. \\ \Omega _2^j &{}= \{ (m,n) | \bar{T}_a(m,n) > t_j \}. \end{array} \end{aligned}$$

From this subsets, contours over them are extracted to define the structures over the decomposed image and by using morphological operators (see [25]), the image is segmented.

## Results and analysis

### Pressure ulcers data base

**Fig. 2:**
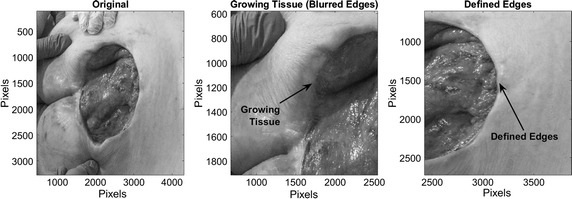
Identified characteristics over a pressure ulcer

To test the technique, a Data Base provided by centre IGURCO containing 51 RGB pressure ulcers images was used. These images are in compression format *JPG* and have a resolution ranging from ${1280 \times 960}$ to ${5184 \times 3456}$. In order to process these images a grayscale convertion and rescaling were conducted. Pressure ulcers over the Data Base were in stage IV with a complete loss of skin and exposed muscle. The images were taken after a debridement process to remove the necrotic tissue. Wounds are characterized by a reddish color and are of different size and shapes. It is also possible that edges that delimit them are blurred. The condition in which the edges are, provide information about the healing process, in this case, if the edges are blurred imply that the tissue is growing, therefore it is necessary to take special care about when conducting the analysis, as it can lead to confuse the necrotic tissue with the healthy. On Fig. 2, an example of a pressure ulcer with defined and blurred edges is shown.

### Tests

**Fig. 3:**
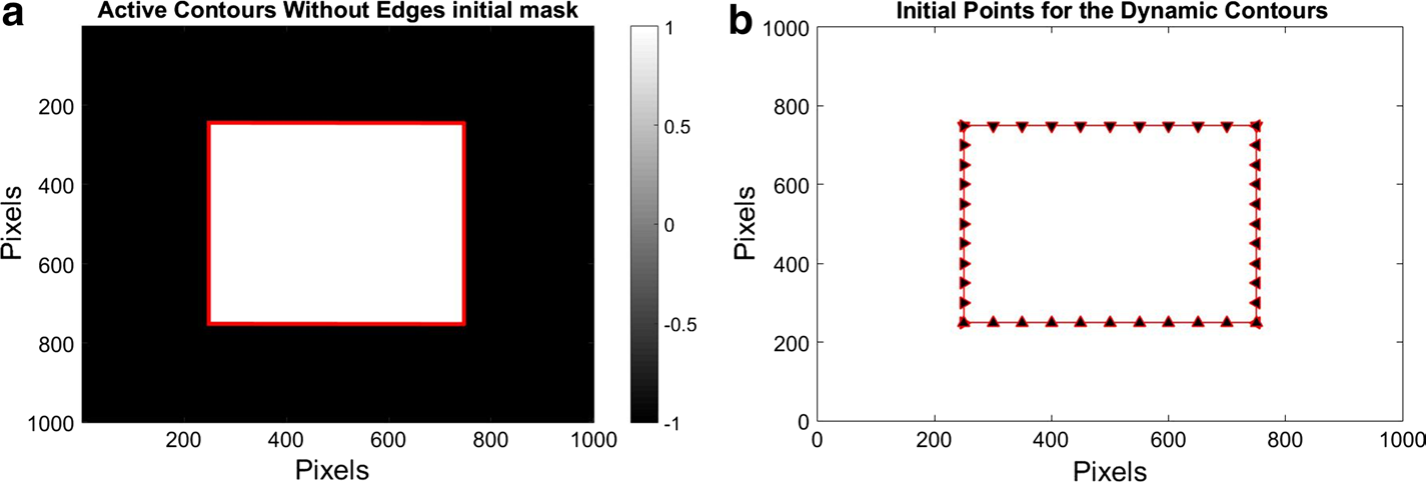
On the *left*, mask for the initial solution of the active contours without edges. The *red line* defines the contour over the function $\phi$ that defines the set $C=\{ (x,y) | \phi (x,y) = 0\}$. On the *right*, initial points for the dynamic contours

**Fig. 4:**
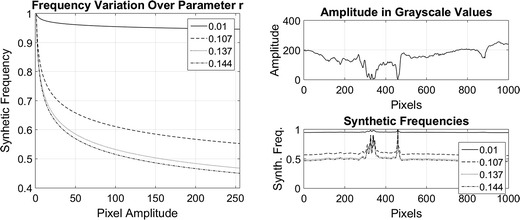
Synthetic frequencies of 4 values of *r* over a row of pixels of an image. On the *left side*, synthetic frequency values for each parameter. On the *right*, synthetic frequencies generated by a row of normalized pixels

**Fig. 5:**
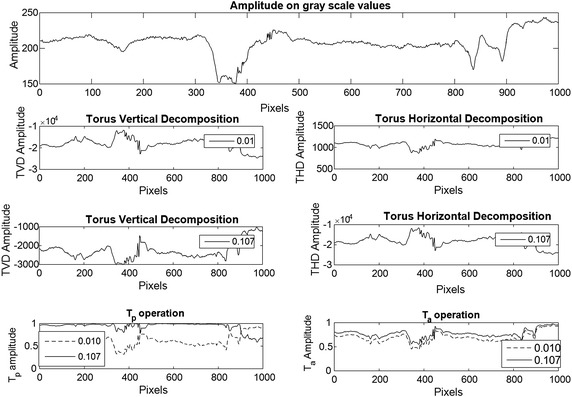
Image decomposition comparison over a row of pixels using 2 values of *r*. On the *top* image, the amplitude on *gray scale* values of a row of pixels over an image is show. The four central images show the torus vertical and horizontal decomposition for two frequency parameter values

In order to analyse the contrast level variations, image decomposition was performed by using different values for the frequency parameter. Here, a row of pixels over the image were taken and the synthetic frequencies for each pixel were calculated using values of 0.01, 0.107, 0.137 and 0.144 for the parameter *r*. These values were selected with the purpose of showing the asymptotic dynamic that have the synthetic frequencies to the variation of this parameter. The values are close to zero by the following analysis of Fig. 1 where for values above 0.5 for difference between a value and the other is not significant. Once the frequency parameters were selected, the torus decomposition was applied. Results are presented in Figs. 3, 4 and 5.

For the image segmentation over the Data Base of pressure ulcers, images were decomposed using 30 levels of contrast variation by changing the parameter *r* of the synthetic frequency (8) ranging from 0.013 to 0.015 by following the analysis of contrast variations. Parameter *R* in (10) was determined experimentally taking into account the image properties in order to reduce the periodicity product of the vertical and horizontal decomposition, in this particular case given the images size and sampling rate the value was set to 100 pixels. To evaluate the proposed technique, images on the Data Base were processed and the results were compared with the respective real segments. Digital image correlation was used as accuracy measure of the algorithm. In this case, correlation is defined as

18 $$\begin{aligned} \rho (A,B) = \frac{\sum _m \sum _n (A_{mn} - \bar{A})(B_{mn} - \bar{B})}{\sqrt{(\sum _m \sum _n (A_{mn}-\bar{A})^2)(\sum _m \sum _n (B_{mn}-\bar{B})^2)}} \end{aligned}$$

where $\bar{A}$ and $\bar{B}$ are the mean value of the image *A* and *B* respectively and *n*, *m* are the pixels positions that goes from 0 to *N* and *M* respectively.

### Algorithm comparison

Results over the proposed technique were compared using the dynamic contours (Snakes) and the active contours without edges (ACWE) proposed in [2, 15], these techniques are based on the definition of a parameterized curve which contains information related with object surface, the domain over this function defines the region of interest from image. The results obtained with all techniques were compared with the real segment using the digital image correlation.

Curve evolution methods have been widely used to segment images. In particular, dynamic contours or snake is a spline which approximate the contour over an image by minimizing an energy functional based on the gradient magnitude of the image. Nonetheless, an initial solution around the object of interest must be provided [13], the curve moves toward the interior and stops when it reaches the contour of the object.

These methods have the capacity of autonomous adaptation but are sensitive to local minima states. Let $\Omega$ be a bounded open subset of $\mathbb {R}^2$ and $\partial {D}$ its boundary. Let ${u_0:\bar{\Omega } \rightarrow \mathbb {R}}$ be a given image and ${C(s,t):[0,1] \times [0,\infty ) \rightarrow \mathbb {R}^2}$ be a parametrized curve with spatial parameter *s* in [0, 1] and temporal variable ${t \in [0,\infty )}$ [26] the evolution of the curve *C*(*s*, *t*) can be expressed as the variational problem $\inf _{C}{J(C;u_0)}$, where $J(C;u_0)$ is defined as

19 $$\begin{aligned} J(C;u_0)=\alpha \int _{0}^{1} |C^\prime (s,t)|^2 ds + \beta \int _{0}^{1} |C^{\prime \prime } (s,t)|^2 ds-\lambda \int _{0}^{1} |\nabla u_0(C(s,t))|^2 ds \end{aligned}$$

where $\alpha$, $\beta$ and $\lambda$ are positive parameters that defines the smoothness of the contour and the movement of the curve [15].

In the ACWE method, the evolution of the curve is given by solving the differential equations with initial condition [15]:

20 $$\begin{aligned} {\left\{ \begin{array}{ll} \frac{\partial \phi }{\partial t} = F|\nabla \phi |\\ \phi (x,y,t)= \phi _0(x,y) \end{array}\right. } \end{aligned}$$

where $\phi (x,y,t)$ is the level set function with the following properties

21 $$\begin{aligned} \begin{array}{ll} \phi (x,y,t) &{}> 0 \quad \text { for } (x,y) \in \Omega \\ \phi (x,y,t) &{}< 0 \quad \text { for } (x,y) \notin \bar{\Omega }\\ \phi (x,y,t) &{}= 0 \quad \text { for } (x,y) \in \partial {\Omega }. \end{array} \end{aligned}$$

In this case the variational problem can be defined using the Heaviside function $H(\phi (x,y))$ as

22 $$\begin{aligned} \min _{\phi ; c_1, c_2 \in \mathbb {R}^+} F&(c_1, c_2, \phi ; u_0); \nonumber \\ F(c_1, c_2, \phi )&= \mu \int _{\Omega } \nabla (\phi (x,y)) |\Delta \phi (x,y)| dx dy \nonumber \\&\quad+ v \int _{\Omega } H(\phi (x,y)) dx dy \nonumber \\&\quad+ \lambda _1 \int _{\Omega } |u_0(x,y)-c_1|^2 H(\phi (x,y)) dx dy \nonumber \\&\quad+ \lambda _2 \int _{\Omega } |u_0(x,y)-c_2|^2 (1 - H(\phi (x,y))) dx dy \end{aligned}$$

where $\mu \geqslant 0$, $\nu \geqslant 0$, $\lambda _1,\lambda _2>0$ are fixed parameters and $c_1, c_2 \in \mathbb {R}^+$ [15].

To the test, the mask presented on Fig. 3 was used as initial solution for the dynamic contours and the active contour without edges. Considering that pressure ulcers can be located in any part of the image, the dimension and shape of the mask was defined in order to cover the maximum possible area so that ulcers can be segmented. This mask represent the signed function $\phi$ with positive values inside, negative outside and its contour defines the set ${C = \{(x,y) | \phi (x,y)=0\}}$. The segmentation is accomplished by redefining the set *C*, using the variational problem formulated. For the dynamic contours, the initial spline is made of 100 point with the same shape used in the mask for the level set. Parameters used for active contours technique are the same as proposed on [2, 15], with values of $\lambda _1 , \lambda _2 = 1$, $\nu = 0$, $\mu =1$ and the step space $\Delta t = 0.1$. For the dynamic contours $\alpha = 0.4$, $\beta = 0.2$ and $\lambda = 1$ used on [2]. The number of iteration where established in 1000.

### Analysis

As it can be seen in Figs. 1 and 4, synthetic frequencies defined in (8) have an asymptotic behaviour, remaining higher near to amplitude value 1. Also, while *r* is closed to 0, synthetic frequencies not exhibit much variation. As the parameter increases, this asymptotic behaviour becomes more significant and the range of frequency values increases. On the bottom right of Fig. 4, synthetic frequencies were generated using a row of pixels over an image. It can be seen that the synthetic frequencies generated with the lower value of *r*, do not present much variation and values are closed to 1. As this parameter increases, the frequency variation changes, appearing an expansion on the values of the frequencies. In this case, the synthetic frequencies generated by pixels of lower amplitude values differ more than those with higher values.

**Fig. 6:**
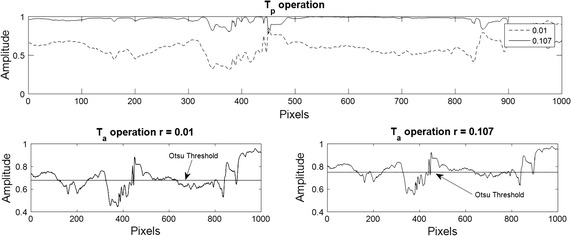
Otsu threshold for 2 values of *r*. The *top* image show the product operation defined on (11). The *bottom* images shown the Otsu threshold for each frequency parameter variation

The asymptotic behavior of the frequencies allows for the variation of the different contrast levels in the processed image. When the frequencies values are replaced on (10), the image is decomposed vertically and horizontally using the *TVD* and *THD* mappings. These mappings change the scale values and invert the contrast over the original image as it can be seen on Fig. 5. Replacing the vertical and horizontal decomposition over the toroidal operations $\bar{T}_a$ and $\bar{T}_p$, threshold level is calculated by using the variational formulation (16). By variating the frequency parameter, amplitude in the toroidal operations changes, generating different thresholds which differentiate high from low gray scale values, as it is shown in Figs. 5 and 6. This differentiation defines the subsets (17) for the correlated data in the image.

**Fig. 7:**
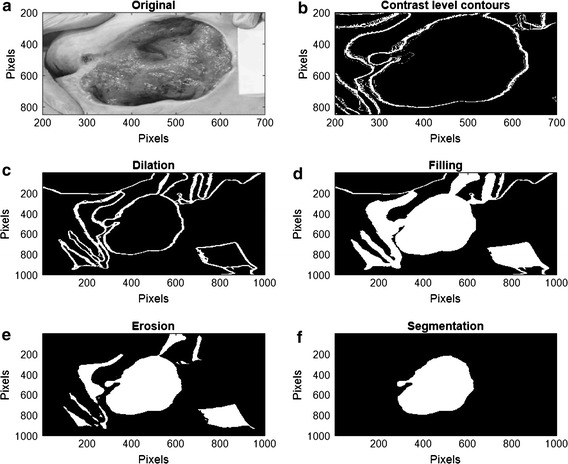
Morphological operation process over the contrast level contours. **a** Is the original image and **b** shows the contours obtain by the image decomposition. **c**–**f** shows the process of dilation and erosion made to segment the image

As it was mentioned before, images over the Data Base were processed using 30 levels of decomposition, with a variation of the frequency parameter between 0.013 and 0.015. This interval was established by analyzing the contrast variations over the torus operations, in order to generate the threshold levels that give information over the image structures. For each level of decomposition, contours of the subsets defined in (17) are extracted as it is shown on the top right of the Fig. 7. In the images, pressure ulcer present low gray scale amplitude values, differentiated as the darkest areas of the image. Here threshold levels cut the image into subset with high and low gray scale values and the contours over them define the edges of the pressure ulcer in the image. Once the contrast levels are defined over the image, the domain of the section of the pressure ulcer is extracted. To achieve this, morphological operations are applied to extract the subset that defines the domain over the wound. On Fig. 7, the process of morphological variations over the contrast levels are shown.

**Fig. 8:**
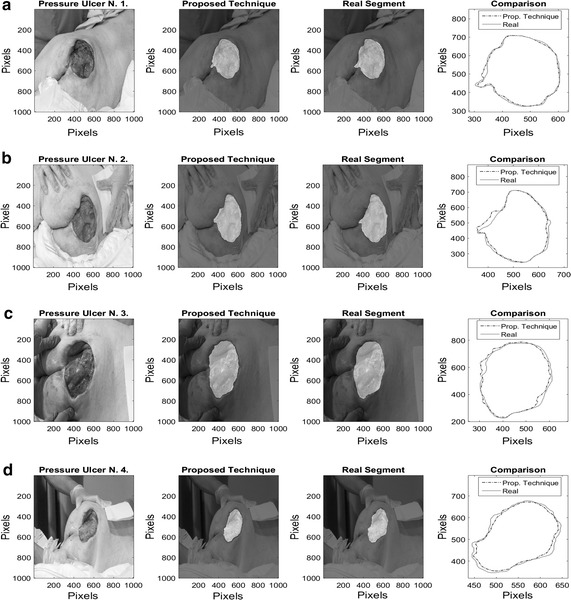
Pressure ulcer segmentation using the proposed technique. Images on the *left column* where segmented using the proposed methodology, and the segment contour is compared with the real one presented on the *last column*

On Fig. 8, results over 4 images of pressure ulcers from the Data Base are presented. Image segmentation using the proposed technique is shown in the second column and the segment contour is compared with the real one presented on the last column. As it can be seen, the proposed technique can describe the segment of the pressure ulcers by detecting the edges over it, even when different kind of objects appear in the images.

**Fig. 9:**
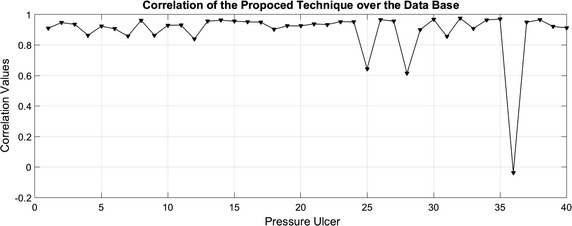
Correlation values over the data base

In this case, segments obtained from the synthesized images define the morphology of ulcers, even in the presence of structures as blurred edges, such as those present on wounds 1 and 3. In addition, Fig. 9 shows the correlation over 40 of the 51 images from Data Base. As it can be seen, images processed with the technique have an average correlation of 0.89 with the real segments of pressure ulcers with a standard deviation of $\pm {0.1}$. From the 40 images, 37 have a higher value of 0.85, which means that the segments detected over the image can describe almost completely the morphology of the pressure ulcer. Although, 11 images within the Data Base were not used because they showed saturation in all the color channels, as a result the gray scale image is also saturated. This saturation avoids the correct calculation of the Otsu’s threshold, since this skews the result to the values of greater intensity in the image. This avoids to define the contrast levels that separate the pixels of the image in the two subsets of (17). In particular, pressure ulcer images 25, 28 and 36 present saturation only in green channel, leading to low correlation with the real segment, for this reason it is proposed as future work to add a pre-processing step in order to enhance the image characteristics and improve the segmentation made by the algorithm.

### Comparison results

**Fig. 10:**
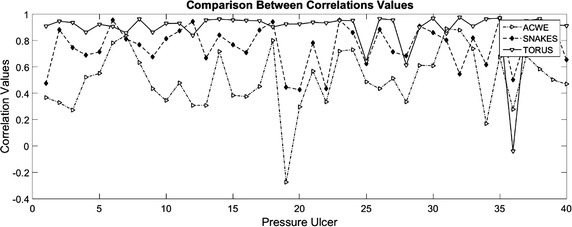
Correlation comparison between of the proposed technique and the benchmarked algorithms

Figure 10 shows the correlation values for the benchmarked algorithms and the proposed technique. In Table 1, statistical results on the correlation values obtained are presented. It can be seen that the proposed technique has better approximation to the real segment with an average correlation values of 0.89, followed by dynamic contours with 0.75 and 0.5 for the active contours without edge. In addition, the Proposed technique has a standard deviation of $\pm {0.1}$, ACWE algorithm have the highest standard deviation value, with $\pm {0.22}$ which means that it is more prone to failure. Correlation values closed to 1 indicates good approximation for the proposed technique, and the standard deviation indicates that is more precise. On the other side, correlation values below 0.85 for the benchmarked algorithms means that they do not approximate correctly to the real segment of the pressure ulcer.

**Table 1:** Statistical results over the correlation values

Method	Mean	Standard deviation	Max	Min	Comp. time (s)
Proposed technique	0.89	0.102	0.977	0.532	9.04
ACWE	0.5	0.226	0.889	−0.274	70.23
Dynamic contours	0.75	0.154	0.967	0.427	78.65

Another important feature is that it is not necessary to give initial solution to the proposed method, contrary to the benchmark algorithms. Considering that the initial solution was generated the same for each of the images, these algorithms are more prone to errors due to a bad initial solution. Besides, these algorithms require a parametrization in order to get a better solution.

**Fig. 11:**
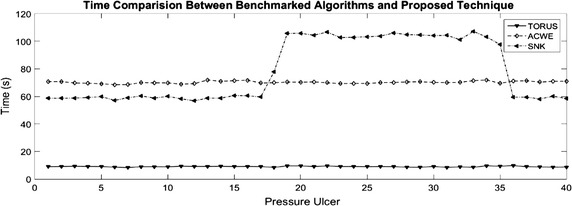
Time comparison between the proposed technique and the benchmarked algorithms

Figure 11, shows the comparison of the computational time for each algorithm. Test were performed using an Intel(R) Core(TM) i7-5500 CPU at 2.40 GHz and images has the same size to minimize time variations. The proposed technique has an average of 9.04s of computational time, the ACWE algorithm of 70.23s and the dynamic contours of 78.65s. The proposed technique exhibits an improved performance against the benchmarked algorithms. From the pressure ulcer 18 to 35, the dynamic contours (Snakes) algorithm present an increased computational time, due to the different objects presented in the images.

**Fig. 12:**
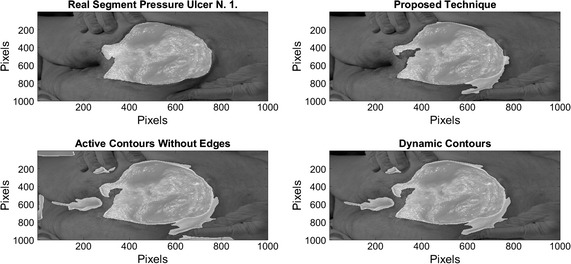
Segmentation results over pressure ulcer N. 1 using the proposed technique (*upper right*) and the benchmarked algorithms (*bottom images*)

**Fig. 13:**
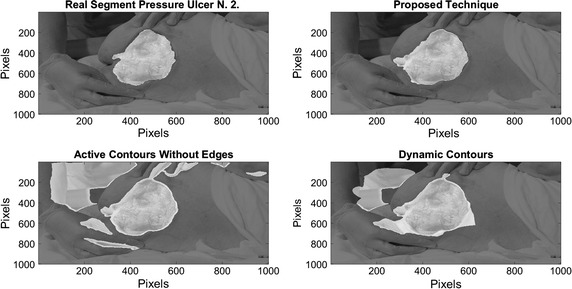
Segmentation results over pressure ulcer N. 2 using the proposed technique (*upper right*) and the benchmarked algorithms (*bottom images*)

**Fig. 14:**
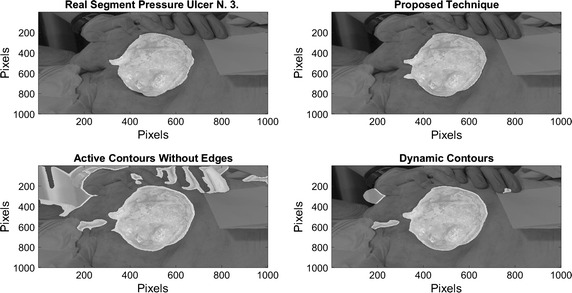
Segmentation results over pressure ulcer N. 3 using the proposed technique (*upper right*) and the benchmarked algorithms (*bottom images*)

**Fig. 15:**
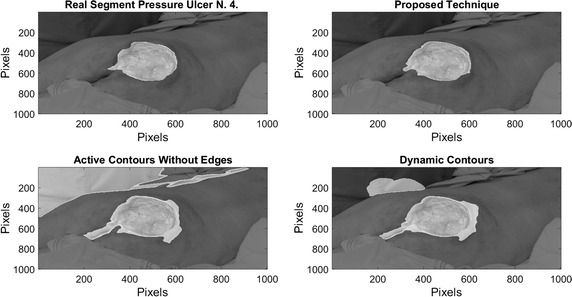
Segmentation results over pressure ulcer N. 4 using the proposed technique (*upper right*) and the benchmarked algorithms (*bottom images*)

**Fig. 16:**
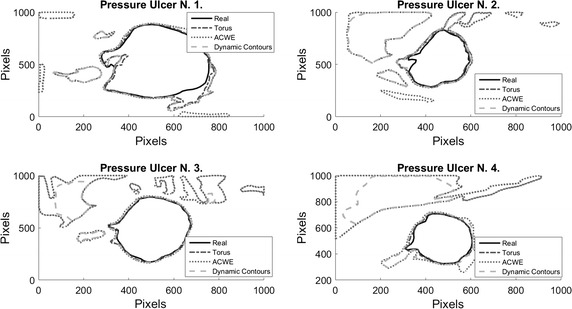
Comparison over the result segmentation by the compared methods

On Figs. 12, 13, 14, and 15 the results related with four pressure ulcer images processing are presented. On the upper left image the real segment of the pressure ulcer is shown, on the upper right image the approximated segment result from the proposed methodology. On the bottom images the result from the active contours without edges and the dynamic contours. Figure 16 presents a comparison over the result segmentation by the compared methods. As it can be seen, the proposed technique shows good approximation to the real segment of the wound but the ACWE and the dynamic contours segment different objects of the image and the edges are more distant from the real contour. Errors in the segmentation using these algorithms may be due to poor selection of parameters and initial conditions.

## Conclusions

The presented technique is a novel segmentation method to extract structures over images. Synthetic frequencies variations give contrast changes using the torus geometry in order to enhance the contrast over the images. The addition and product operations, that depend of the synthetic frequencies over the vertical and horizontal decomposition, weights amplitudes in the image and by using the Otsu method it is possible to define structures over the original images. Using morphological operations, this scheme of transformation allows to relate the spatial information with the contrast of the images.

This transformation scheme allows to select intervals of contrast variation making feasible to work with specific areas of the images where the contrast levels are similar. In addition, as the operations to calculate the contrast variation does not depend on the neighbourhood, it is possible to avoid complex operations as convolution, allowing to use the technique locally in the image. As the system uses the information over the image, is not necessary to give an initial solution, opposite to the other algorithms, where an initial solution must be established. This is an advantage when processing large amounts of pictures, because there is not necessary prior knowledge of the image to process.

Tests were performed over pressure ulcer images provided by the Centre IGURCO in order to extract the spatial information of the lesion. The proposed technique shows good results over the used images even in presence of artifacts as noises and objects used by the medical personnel when conducting the evaluation. The morphological characteristics over this spatial information, provides useful information for eventual diagnosis. As the presented technique is based on the contrast variation, saturation over the color channels can lead to errors over the segmentation. As future work it is proposed to design a methodology that includes a step of preprocessing, in order to remove the saturation that reduce the performance of the technique.

Some tests were conducted in order to compare the proposed technique with benchmark algorithms. Considering that these algorithms require an initial condition and a previous parametrization, the developed technique present an advantage from the generalization of the solutions on the images. In addition, computational time in conjunction with accuracy and precision, exhibit a good methodology for image segmentation of pressure ulcer, with the objective to conduct parametrization of the morphology of the wound.

## Abbreviations

NPUAP: National Pressure Ulcer Advisory Panel
TVD: toroidal vertical decomposition
THD: toroidal horizontal decomposition
ACWE: active contours without edges
